# Viruses of the *Turriviridae*: an emerging model system for studying archaeal virus-host interactions

**DOI:** 10.3389/fmicb.2023.1258997

**Published:** 2023-09-21

**Authors:** Michael S. Overton, Robert D. Manuel, C. Martin Lawrence, Jamie C. Snyder

**Affiliations:** ^1^Department of Biological Sciences, Cal Poly Pomona, Pomona, CA, United States; ^2^Division of Biological Sciences, University of California, San Diego, La Jolla, CA, United States; ^3^Department of Chemistry and Biochemistry, Montana State University, Bozeman, MT, United States

**Keywords:** archaea, virus, Sulfolobus turreted icosahedral virus, virus-host interaction, viral replication, *Turriviridae*

## Abstract

Viruses have played a central role in the evolution and ecology of cellular life since it first arose. Investigations into viral molecular biology and ecological dynamics have propelled abundant progress in our understanding of living systems, including genetic inheritance, cellular signaling and trafficking, and organismal development. As well, the discovery of viral lineages that infect members of all three domains suggest that these lineages originated at the earliest stages of biological evolution. Research into these viruses is helping to elucidate the conditions under which life arose, and the dynamics that directed its early development. Archaeal viruses have only recently become a subject of intense study, but investigations have already produced intriguing and exciting results. STIV was originally discovered in Yellowstone National Park and has been the focus of concentrated research. Through this research, a viral genetic system was created, a novel lysis mechanism was discovered, and the interaction of the virus with cellular ESCRT machinery was revealed. This review will summarize the discoveries within this group of viruses and will also discuss future work.

## 1. Introduction

As they have become more widely studied, Archaea have only become more interesting, exceptional, and immediately relevant, and this is no less true for their viruses. Despite this increasing interest, which has led to the identification of 18 archaeal viral families (a majority infecting the Euryarchaeota and Crenarchaeota), the study of archaeal viruses continues to be overshadowed by the viruses that infect the other two domains of life (Dellas et al., 2014; Krupovic et al., 2018; Munson-McGee et al., 2018). This is somewhat perplexing, since viruses infecting archaeal hosts display such novelty in their morphologies and infection cycles. Archaeal virus capsids run a spectrum from rigidly symmetric geometries in *Turriviridae* (Rice et al., 2004) and *Sphaerolipoviridae* (Jaakkola et al., 2012), to the more fluid forms (Dharmavaram et al., 2018), such as the lemon or spindle-shaped viruses of the *Fuselloviridae* (Han et al., 2022) and *Bicaudaviridae* (Hochstein et al., 2018; Wang et al., 2022), the bottle shaped *Ampullaviridae* (Häring et al., 2005), the ovoid *Guttaviridae* (Arnold et al., 2000; Mochizuki et al., 2011), and the pleomorphic *Pleolipoviridae* (Pietilä et al., 2016; Demina and Oksanen, 2020). Viruses infecting the Crenarchaeota often exhibit unique morphologies yet to be identified anywhere else in nature (Lawrence et al., 2009; Dellas et al., 2014; Snyder et al., 2015; Baquero et al., 2020). In contrast, viruses infecting the Euryarchaeota frequently exhibit morphologies similar to those of tailed bacteriophages (Atanasova et al., 2012; Dellas et al., 2014; Baquero et al., 2020).

Genetically, the viruses of archaea are highly divergent from those of the other domains and from each other (Prangishvili et al., 2006; Dellas et al., 2014). Still, the few homologs discovered so far have provided powerful insights into the mechanisms, functions, and origins of viral systems across the domains (Birkenbihl et al., 2001; Blum et al., 2001; Peng et al., 2001; Khayat et al., 2005; Krupovic et al., 2018). In addition, structural and functional characteristics of viral proteins have linked biological processes such as cell division, trafficking, and transcriptional regulation (Iyer et al., 2004; Aravind et al., 2005; Snyder et al., 2013b).

Some of the best studied archaeal viruses infect members of the *Sulfolobaceae* family. Members of this archaeal family typically inhabit high temperature, low pH environments and include, among others, the genera *Sulfolobus*, *Acidianus*, and *Metallosphaera* (Albers and Siebers, 2014). *Sulfolobus shibate* virus 1 (SSV1) was the first virus infecting a *Sulfolobus* species to be isolated (Palm et al., 1991; Schleper et al., 1992). Since that time, there have been several other viruses isolated from *Sulfolobus* and other *Sulfolobaceae* (Prangishvili, 2013; Dellas et al., 2014; Krupovic et al., 2018). In addition, several metagenomic studies have focused on viruses infecting this family (Schoenfeld et al., 2008; Gudbergsdóttir et al., 2015; Hochstein et al., 2016; Anderson et al., 2017; Liu et al., 2019). Over the years, three virus families infecting *Sulfolobus* species have developed into model systems for studying archaea and archaeal viruses: *Fuselloviridae* (SSV-like viruses), *Rudiviridae* (SIRV-like viruses), and *Turriviridae* (STIV-like viruses) (Lawrence et al., 2009; Prangishvili, 2013; Dellas et al., 2014; Krupovic et al., 2018; Munson-McGee et al., 2018). This review will focus on what has been learned about archaeal viruses and archaea by studying the *Turriviridae*.

## 2. STIV1

### 2.1. Discovery, isolation, and initial characterization

*Sulfolobus* turreted icosahedral virus (STIV) was originally detected in samples collected from Midway Geyser Basin in Yellowstone National Park, United States (Rice et al., 2001, 2004). The virus-like particle (VLP) with icosahedral morphology was isolated from two enrichment cultures and double-stranded DNA (dsDNA) was extracted from the particles (Rice et al., 2001). Sequencing of these VLPs did not immediately reveal any relationships to known viral genomes, but 16S rRNA sequencing of the host identified it as a close relative of *S. solfataricus* (Rice et al., 2001, 2004). Initial characterization of the virus’ structure revealed striking morphological features, inspiring its name, *Sulfolobus* Turreted Icosahedral Virus (STIV). We have recently renamed this virus to STIV1 since other variants have been isolated and studied (discussed below).

After sequencing the 17,663 bp circular dsDNA genome, the 36 putative open reading frames (ORFs; since updated to 38 ORFs) appeared to be a mix of a few conserved genes present in other *Sulfolobus* viruses and a large fraction of completely novel sequences (Rice et al., 2001; Maaty et al., 2012a). Only four of these ORFs, B116, B204, C92 and C557, produced matches to other known sequences when initially searched in nucleotide and protein databases (Rice et al., 2004). Further structural and biochemical studies have elucidated several other STIV genes with known homologs in other virus families and even other organisms, which are discussed below.

### 2.2. The STIV1 virion structure reveals ancient viral relationships

**Figure 1:**
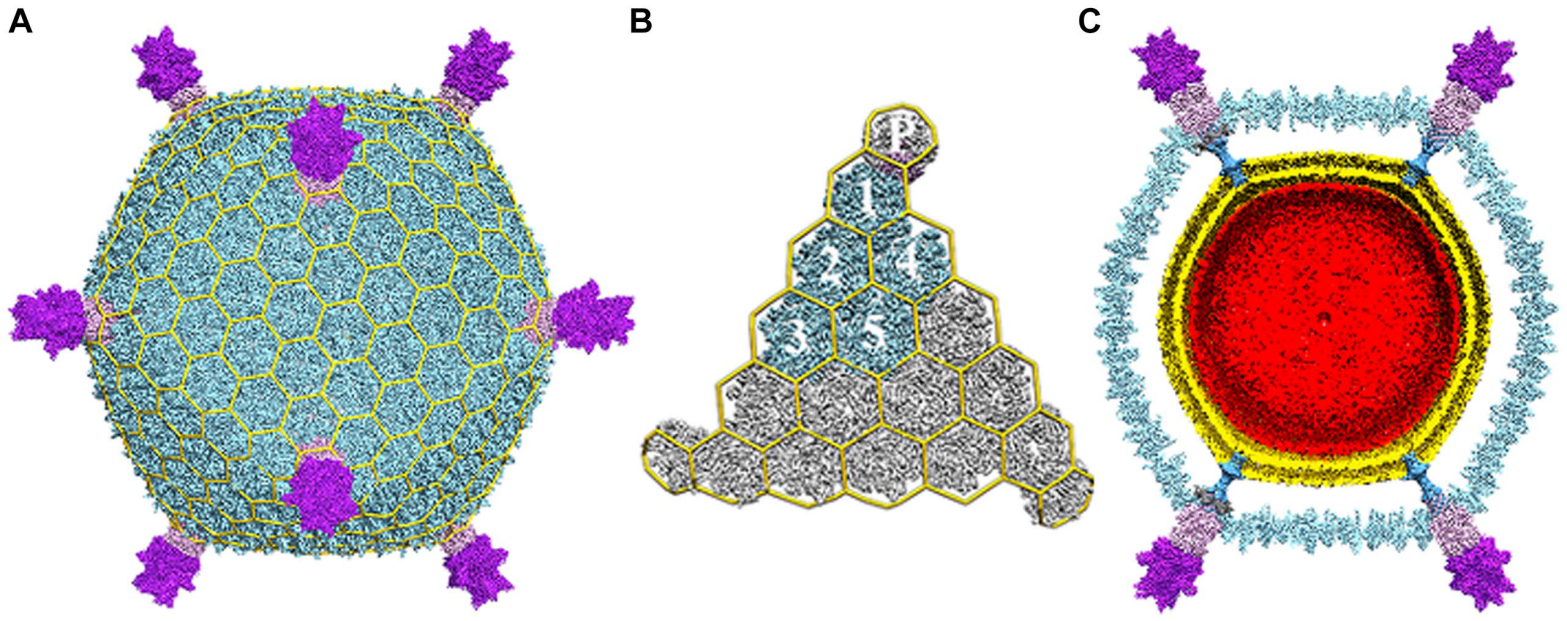
Electron cryo-microscopy reconstruction of STIV. **(A)** The overall virus reconstruction is displayed with the different protein components individually colored (B345: light blue, A223: light pink, C381: purple) and with an icosahedral cage overlaid onto it. **(B)** Blow-up view of an icosahedral face with one capsid icosahedral asymmetric unit colored as in **(A)** and labeled (1–5 for the trimeric B345 capsomers and *P* for the A223 penton base). **(C)** Cross-section of the reconstruction revealing the presence of internal lipid envelop (gold) and the internal genome (red). Adapted from Veesler et al. (2013).

**Figure 2:**
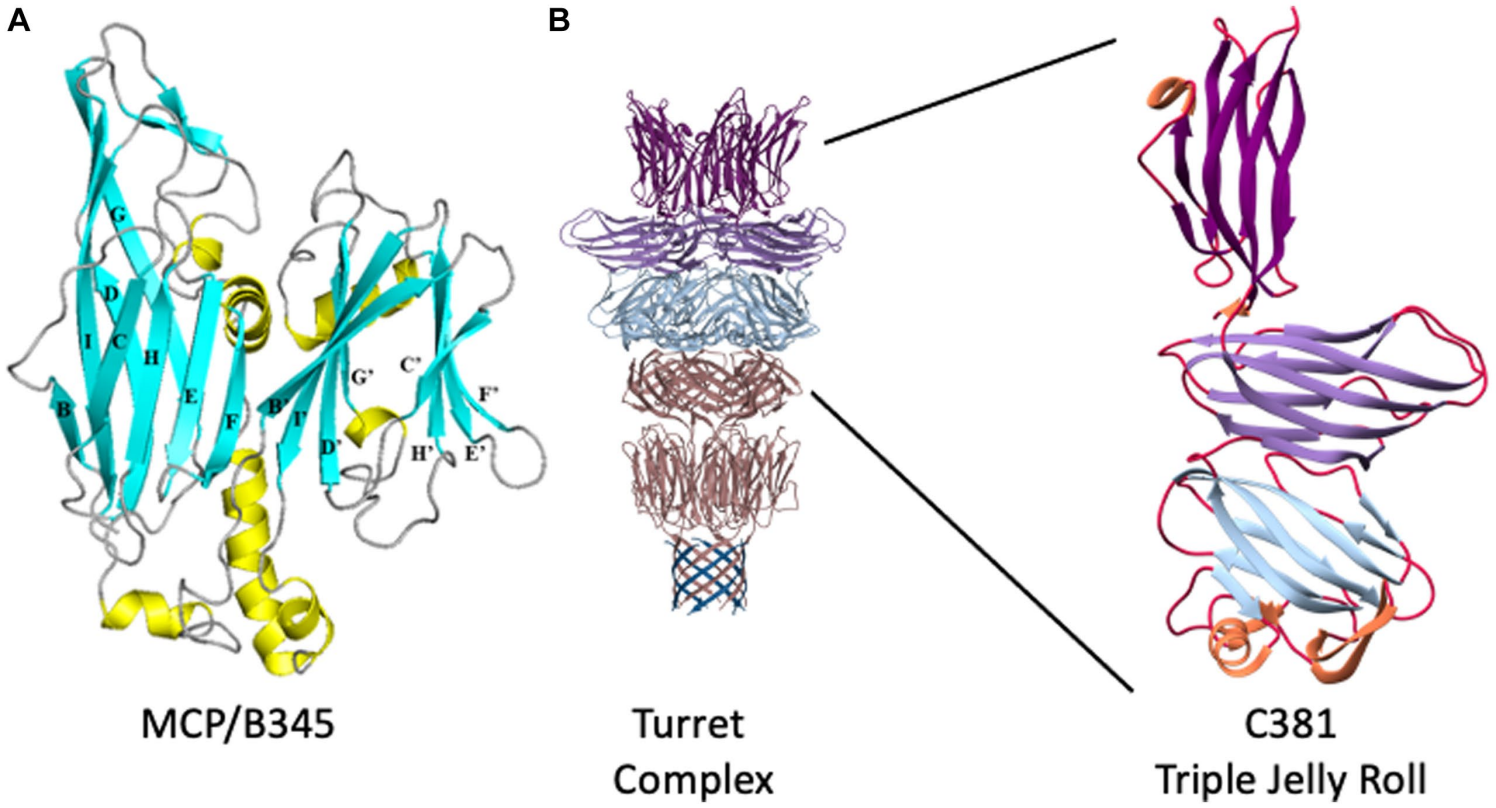
STIV structural proteins. **(A)** Ribbon representation of B345, the major capsid protein of STIV. *β*-strands are depicted in cyan, *α*-helices in yellow, loops in gray. The structure is composed of two domains, an N-terminal domain (left) and a C-terminal domain (right). Each domain adopts the jelly roll fold, which is composed of two four-stranded *β*-sheets. In the N-terminal domain, the first sheet is formed of strands B, I, D and G (BIDG) at the back of the structure, and the second sheet of strands C, H, E and F (CHEF) at the front of the structure. The two β-sheets pack against each other to form a *β*-sandwich. Similarly, the C-terminal jelly roll is composed of strands B′, I′, D′, and G’ in the B’I’D’G’ sheet, and strands C′, H′ E’, and F′ in the C’H’E’F′ sheet. Adapted from Khayat et al. (2005). **(B)** The structure of the STIV turret. *β*-strands emanating from the A55 membrane anchor are at the bottom, in blue, where they interdigitate with the N terminus of A223 to form a ten-stranded hemolysin-like *β*-barrel. The two-domain, double jelly roll structure of A223 is shown above that in pink, followed by the three-domain jelly roll structure of C381 in light blue, light purple, and dark purple at the top. To the right, an enlarged view of a single C381 protomer. The N-terminal jelly roll is at the bottom, the C-terminal jelly roll domain at the top. Adapted from Hartman et al. (2019).

The structural characterization of STIV1 (Figure 1), carried out by cryo-Electron Microscopy (cryo-EM) and X-ray crystallography has provided substantial insight into the evolutionary history of STIV and large dsDNA viruses in general (Rice et al., 2004; Khayat et al., 2005, 2010; Fu et al., 2010; Veesler et al., 2013; Hartman et al., 2019). Specifically, structural studies of the major capsid protein (MCP, B345) identified a double jelly roll structure (Figure 2A). This double jelly roll fold is also found in eukaryotic viruses, such as adenoviruses and the fungal virus PBCV-1, as well as bacteriophage such as PRD1 (Benson et al., 1999; Nandhagopal et al., 2002; Fabry et al., 2005). The discovery of the double jelly roll fold in STIV thus extended observation of this viral capsid architecture to all three domains of life, and led to realization that structural similarities in the “viral self” imply common viral ancestry (Bamford, 2003; Bamford et al., 2005; Khayat et al., 2005; Abrescia et al., 2012). Critically, sequence similarities among disparate viruses whose major capsid proteins exhibit the double jelly roll fold can be quite low, indicative of extremely distant evolutionary relationships that potentially predate the last universal common ancestor (LUCA) of cellular life (Rice et al., 2004; Khayat et al., 2005). For example, the STIV major capsid protein shows only 8% identity to the PBCV-1 major capsid protein, only marginally higher than if it were aligned with a random protein sequence (1/20 = 5%).

Each of the jelly roll domains is composed of eight β-strands arranged in two, four-stranded antiparallel β-sheets, historically denoted as “BIDG” and “CHEF” (Figure 2A). The two *β*-sheets are packed against each other to bury a hydrophobic interface (Khayat et al., 2005). Within the capsid, B345 forms trimers that, because of the double jelly roll, appear as pseudo-hexamers. These pack into triangular faces surrounding the five-fold vertices to instantiate the complete capsid geometry (Rice et al., 2004; Khayat et al., 2005; Maaty et al., 2006). The capsid lattice geometry of large dsDNA viruses is quite varied (Montiel-Garcia et al., 2021) and STIV is no exception, adopting a previously undescribed pseudo-T = 31d icosahedral lattice (Figures 1A,B; Rice et al., 2004). In this lattice geometry, each triangular face is composed of three asymmetric subunits, with each subunit composed of five MCP trimers (hexons) joined obliquely with the turret vertex structure (Rice et al., 2004). The icosahedral subunit thus contains 15 copies of B345; as well as one copy each of A223, C381 and A55, each arranged around the five-fold vertex.

While the structure of the capsid is fascinating on its own, one of the truly emblematic features of the STIV1 virion are the large turret-like structures protruding from each of the 12, five-fold vertices (Figures 1, 2). These turrets extend approximately 12 nm above the capsid shell, or ⅓ of the 36.5 nm capsid radius (Rice et al., 2004; Khayat et al., 2005; Fu et al., 2010; Veesler et al., 2013). Each turret is built from a C381 pentamer stacked on top of an A223 pentamer. Like B345, A223 is also composed of two jelly roll domains fused within a single polypeptide chain. The N-terminal jelly roll interfaces with the capsid shell, where it forms the foundation of the turret, or “penton base.” The second domain is positioned above the surface of the capsid and forms the first floor of the turret. The N-terminus of C381 interfaces with A223, and its “triple jelly roll” fold forms the second, third and fourth floors of the turret (Figure 2B; Veesler et al., 2013; Hartman et al., 2019). The jelly roll domain is thus ubiquitous in the structural proteins of STIV1. In addition to interactions with the major capsid and turret proteins (B345, C381), the N-terminal *β*-strands of the A223 pentamer reach into the interior of the capsid, where they interdigitate with *β*-strands from A55, forming a 10-stranded antiparallel, hemolysin like *β*-barrel as the basement of the turret. A55, in turn, is anchored within the internal lipid envelope by a transmembrane *α*-helix. A55 thus forms a sub-basement for the turret (Figure 2B), and the A223/A55 interactions anchor the capsid to the internal lipid membrane.

In an early single particle analysis of STIV1, each turret was decorated by a set of five large, looped petal-like structures, giving the turret a flower-like appearance, 20 nm in diameter (Khayat et al., 2005). Each petal is composed of a single protein, thought to be encoded by the ORF C557 (Rice et al., 2004; Khayat et al., 2005; Maaty et al., 2006). The N-terminus of C557 contains an ankyrin repeat and YLP repeat motifs, both of which are involved in protein–protein interactions (Maaty et al., 2006), while the proline and serine rich C-terminus has no predicted motifs.

The center of each turret contains a 3 nm channel, just wider than a linear dsDNA molecule (2.3 nm), and so could conceivably serve as a conduit between the interior and exterior of the virion, which is discussed below (Rice et al., 2004). Like STIV, adenovirus (Henry et al., 1994), PRD1 (Huiskonen et al., 2007) and PBCV-1 (Shao et al., 2022) also incorporate “penton proteins” that form “spikes” or “filaments” emanating from the five-fold vertices. In each case these are thought to be involved in host recognition and viral entry.

### 2.3. An internal lipid envelope

Though external lipid envelopes are more common, a number of viruses incorporate internal lipid envelopes (Butcher et al., 2011), including the aforementioned PBCV-1 (Nandhagopal et al., 2002) and PRD1 (Bamford et al., 1995; Butcher et al., 1995). While the function of the inner envelope of STIV has yet to be fully elucidated, in addition to a permeability barrier, it also appears to play a unique role in virion assembly, and may serve other functions as well (Rice et al., 2004; Fu et al., 2010). Specifically, cryogenic electron tomography (CET) suggests the envelope is assembled within the cytoplasm, where it may mediate capsid assembly by recruiting and organizing structural proteins at the nascent membrane surface (Maaty et al., 2006; Fu and Johnson, 2012). The structure of the turrets provides additional support for this capsid-membrane co-assembly model (Veesler et al., 2013). The turrets, built from proteins A223, C381 and A55, are the strongest points of contact between the capsid and the underlying membrane (Figure 1C). The association of the membrane protein (A55) with the penton base (A223) may promote nucleation and subsequent growth of the capsid shell (Veesler et al., 2013). In addition, the MCP has a hydrophobic C-terminal helix confirmed to interact with membrane lipids (Khayat et al., 2005, 2010; Fu et al., 2010). Indeed, the partially assembled virions imaged by Fu et al. (2010) appear to be composed of a half sphere of membrane/capsid structure, rather than a fully formed vesicle onto which the capsid components could be recruited.

The externally enveloped *Sulfolobus islandicus* filamentous virus 1 (SIFV1) and ovoid-shaped *Sulfolobus* ellipsoid virus 1 (SEV1) are also thought to acquire their envelopes intracellularly (Wang et al., 2018; Baquero et al., 2021). Because the STIV and SIFV membranes are enriched with a subpopulation of host membrane lipids and internal membrane-bound compartments have never been observed in *Saccharolobus* or any other archaeal cells, budding through an intracellular membrane or vesicle-hijacking seem unlikely (Maaty et al., 2006; Baquero et al., 2021). Instead, envelopment is thought to occur by *de novo* membrane formation or trafficking of lipids from the cytoplasmic membrane to the virion assembly centers. This novel mechanism for cytoplasmic envelopment might be a common feature of these evolutionarily unrelated archaeal viruses (Baquero et al., 2021).

### 2.4. Glycosylation of the major coat protein

Structural analyses of other genes expressed during the infection process have been performed as well (Table 1). For example, X-ray crystallographic studies of A197 revealed a GT-A fold, conserved across a diverse super-family of glycosyl transferases (Larson et al., 2006). Interestingly, the 3-D structure of A197 most closely resembled glycosyl transferases in eukaryotes, and humans in particular. This despite sharing only 15% sequence identity (Larson et al., 2006). There are many examples of viruses decorating self and host proteins with sugars to regulate interactions with their hosts. In this context, Maaty et al. found the STIV major capsid protein, B345, is also glycosylated (Maaty et al., 2006). This suggests STIV may encode its own glycosyl transferase in order to glycosylate B345 during viral assembly in the cytoplasm, a reaction that presumably utilizes a donor substrate produced by cellular glycosylation pathways. However, the true identity of the donor and acceptor substrates remains to be determined (Larson et al., 2006; Maaty et al., 2006).

**Table 1:** Structural and mutational studies of STIV1 ORFs.

STIV1 ORF	Genetic analysis	Structural analysis	Conclusions
B345 (MCP)	Insertion of Ser within a short loop region at residue 223: no viral genomes detected of 22 residues from C-terminal tail: viral genomes not detected (1)	Structure consists of a double jelly roll and C-terminal transmembrane *α*-helix, each jelly roll is composed of eight antiparallel *β* strands (2); glycosylation is essential for replication (3)	B345 loop region integral to protein structure; C-terminal tail is likely integrated into the internal lipid membrane; B345 may be evolutionarily related to MCPs of other viruses, such as adenoviruses, PBCV-1, and PRD1
A223	N/A	As with B345, tertiary structure is a double jelly roll, one domain interfaces with B345, the other is stacked above, forming the base of the turret (4)	As a pentamer, forms the base of the turret structure; associates with A55 in the internal membrane, anchoring the turret and supporting the capsid
C381	Knock-out (premature stop codon via frameshift): viral genomes not detected (1)	Forms a triple jelly roll, each with a different orientation (2, 4); stacks onto A223 and forms the top of the turret (4); the outward lateral cleft formed by domains 2 and 3 binds to host pili (5)	The upper portion of each turret structure is composed of a C381 pentamer; binds to host pili and facilitates viral entry
A55	N/A	C-terminal transmembrane helix associates with virion internal membrane, N-terminal *β*-sheet forms a 10-stranded *β*-pore with A223 (4)	A55 anchors the turret structure to the internal membrane, stabilizing the capsid; along with A223, may nucleate capsid/membrane co-assembly
C557	N/A	Temporarily binds to the outward lateral face of domains 1 and 2 of C381, likely during assembly and exit from host (2, 3, 6);	Exact function unknown, but may block C381 from prematurely attaching the virion to the spent host cell during exit
B204	Effects on ATP hydrolysis of mutations targeting each identified domain: most abolished activity, some tolerance in Arginine finger and ATP-sensing and tunnel residues (7)	Member of the A32-like DNA-packaging clade within the FtsK/HerA superfamily, Walker A and B motifs; forms a hexameric ring with a basic residue-rich internal face (7)	Forms a unique portal vertex that translocates the linear viral genome into the pre-assembled capsid shell, powered by ATP hydrolysis
A197	D151E point mutation: delayed viral replication, knock-out (premature stop codon via frameshift) or deletion: viral genomes not detected (1)	Structure is highly homologous to several glycosyltransferases exhibiting the GT-A fold, no additional functional domains (8)	A197 is an essential glycosyltransferase; may define the minimal structure necessary for activity; possibly glycosylates B345
C92	Knock-out (premature stop codon via frameshift): viral genomes not detected (9)	No direct structural studies. Structural prediction of homolog P98 in SIRV2 yields four α-helices, one of which is likely transmembrane (10)	C92 is the protein component of lysis pyramid structures; monomers polymerize during association with host membrane and deform it; facets separate by unknown mechanism to release mature virions
F93	N/A	Homodimer with interchain disulfide bond, member of MarR-like family of wHTH DNA-binding proteins (11)	Lack of auxiliary domains suggests F93 adopts a constitutively active regulatory state
B116	Deletion mutant: delayed viral replication (1)	Saddle-shaped homodimer; nucleic acid binding domains and DNA binding activity (12)	No specific DNA target defined; may regulate host type III CRISPR-Cas system rather than STIV transcription

MCP, major capsid protein.

References: ^1^Wirth et al. (2011), ^2^Khayat et al. (2005), ^3^Maaty et al. (2006), ^4^Veesler et al. (2013), ^5^Hartman et al. (2019), ^6^Khayat et al. (2010), ^7^Dellas et al. (2016), ^8^Larson et al. (2006), ^9^Snyder et al. (2011b), ^10^Quax and Daum (2018), ^11^Larson et al. (2007a), ^12^Larson et al. (2007b).

### 2.5. Genome packaging

**Figure 3:**
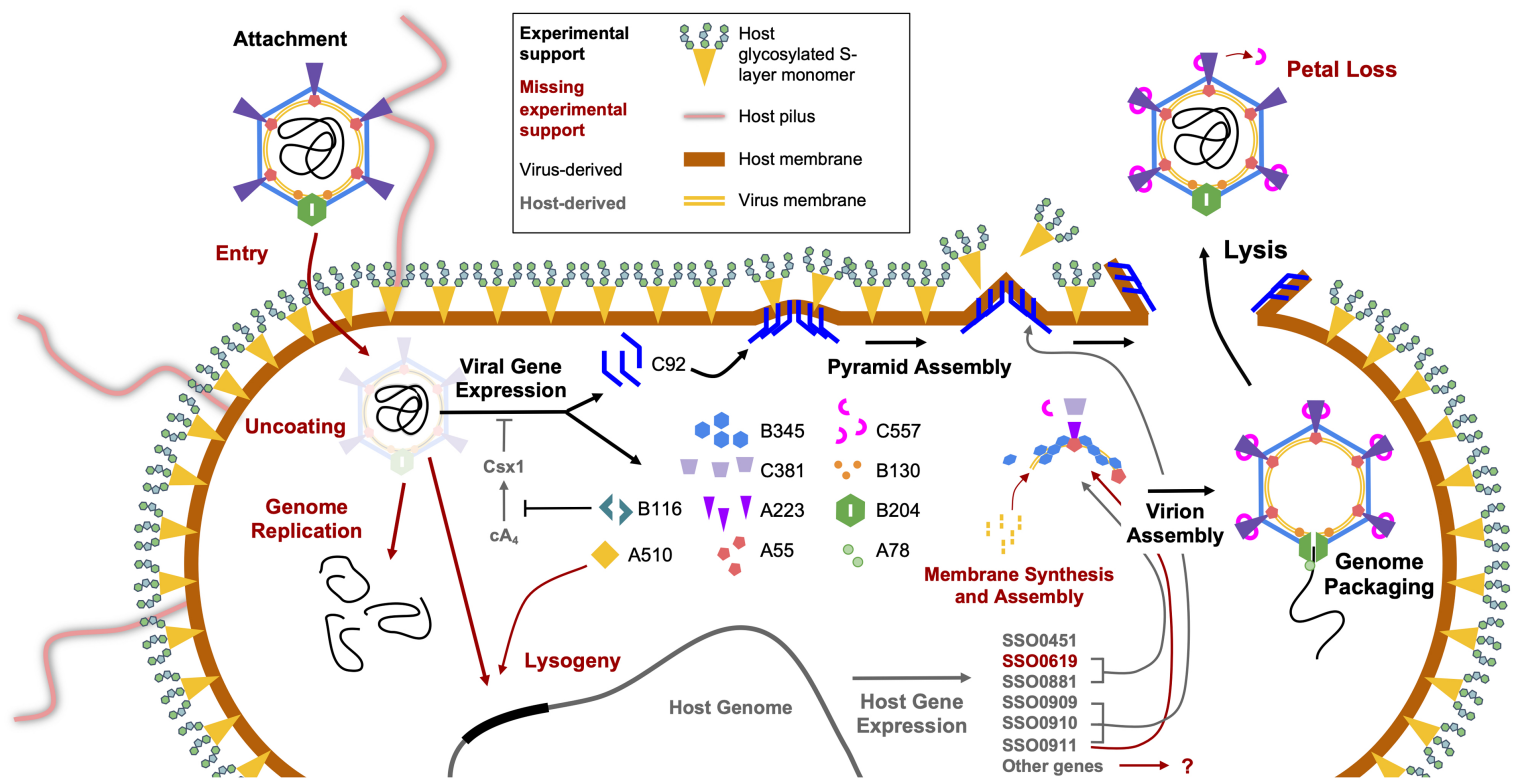
Replication cycle of STIV. The several steps involved in host infection by STIV are shown, starting with viral attachment in the top left and ending with viral egress at the top right. Those steps that have been experimentally characterized are indicated by large black arrows and text, and those yet to be characterized are in dark red. The names of gene products originating from the virus are in black and those from the host are in grey. Smaller black and grey arrows indicate the involvement of virus and host gene products, respectively, in various processes of infection, and red arrows indicate hypothesized involvement.

STIV1 also encodes an ATPase (B204; Dellas et al., 2016) that is thought to drive the packaging of the viral genome (Figure 3). While structural studies have yet to observe B204 in association with purified virions (Rice et al., 2004; Veesler et al., 2013), biochemical work has demonstrated that it is a component of the virion structure (Maaty et al., 2006). Further, two distinctive STIV particles have been isolated from infected cells. One possesses B204 (via western blot), ATPase activity (via *in vitro* assay) and packaged genomes (via qPCR); the other does not. These likely correspond to the filled and empty capsids observed in other studies (Fu et al., 2010; Dellas et al., 2016). There is further evidence that STIV follows the DNA packaging mechanism of the PRD1 lineage of viruses in its sister variant, STIV2 (Strömsten et al., 2005; Hong et al., 2014). The B204 ATPase from STIV2 has also been structurally and biochemically characterized, which has confirmed its presence in the viral capsid and role in packaging the viral genome (Happonen et al., 2013).

### 2.6. STIV1 has an extraordinary host lysis system

**Figure 4:**
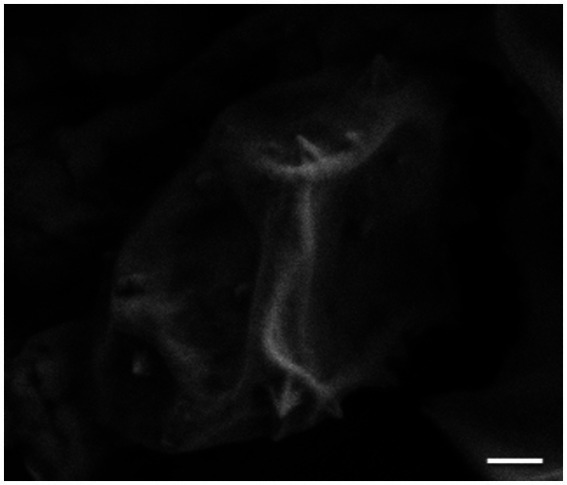
Scanning electron micrograph (SEM) image of STIV1-induced pyramids on the surface of an infected *Sulfolobus* cell. Scale bar = 200 nm.

Looking beyond the virion itself, the structures of other viral proteins have been elucidated, with some striking results. One of the most exciting discoveries in archaeal virology was that of the STIV1 lysis pathway, which involves the eruption of seven-faced, proteinaceous pyramidal structures through the host cell envelope (Figures 3, 4; Brumfield et al., 2009; Snyder et al., 2011b, 2013a). The vertices of these membrane-protein hybrid structures then unzip, allowing the triangular faces to fold out and form hollow “flowers” through which the virions can escape. The pyramids are formed by a single virus-encoded protein, a mere 92 amino-acids in length (C92), that assembles within the cell membrane (Snyder et al., 2011b). It is unclear exactly how the C92 subunits achieve such a multitude of functions, from spontaneous assembly into protein-membrane sheets to the oblique forces that deform the cell envelope outward, to the secondary alignment of C-terminal tails at the pyramid edges, to the tensive and rotational forces that likely catalyze its release (Quax et al., 2011; Snyder et al., 2011b, 2013a; Daum et al., 2014; Quax and Daum, 2018). As the membrane/C92 panels pierce through the S-layer from the inside out, the latter dissociates away, leaving only the pyramid. Finally, the pyramid facets dissociate and open like flower petals, creating a portal for viral egress (Figure 3; Brumfield et al., 2009; Snyder et al., 2011b). It has been hypothesized that host or other viral factors and/ or signals are involved in the opening of the pyramid structures (Snyder et al., 2013a), but this has not yet been experimentally confirmed.

This process is in stark contrast to the holin/endolysin system common in bacteriophages, in which holin aggregates and forms toroidal pores in the membrane, releasing endolysin into the cell wall to proteolytically degrade the peptidoglycan polymers until cellular rupture (Young, 1992, 2014; Catalão et al., 2013). A C92 homolog, P98, was synchronously discovered and characterized in an evolutionary unrelated archaeal virus, *Sulfolobus islandicus* rod-shaped virus 2 (SIRV2) (Bize et al., 2009; Quax et al., 2010, 2011; Daum et al., 2014). Both of these proteins readily form pyramidal structures in the cell membranes of not only members of the *Sulfolobus* genus, but also *E. coli* and *Saccharomyces cerevisiae*, and so in organisms from all three domains of life (Quax et al., 2011; Snyder et al., 2011b; Daum et al., 2014). C92 homologs have been discovered in metagenomic sequences from environmental samples as well, expanding the known diversity of this gene. Incredibly, four distinct clades carrying C92 homologs were found to coexist in a single geothermal region, with diversity spanning that between STIV and SIRV-like viruses (Snyder et al., 2011a). In addition to the virus-induced pyramids of STIV1 and SIRV2, two other archaeal viruses, SEV1 and SIFV1, construct pyramids in the cell envelopes of their hosts, *Sulfolobus* species A20 and *Sulfolobus islandicus*, respectively (Wang et al., 2018; Baquero et al., 2021). These virus-induced pyramids are like STIV1 and SIRV2 induced pyramids in that they open to form polygonal apertures on the cell surface (Wang et al., 2018; Baquero et al., 2021). However, both SEV1 and SIFV1 induced pyramids are six-sided. The pyramids produced by SIFV1 infection are the result of one viral protein, gp43 (an 89 amino acid protein), which is distinct from C92/P98 (Baquero et al., 2021). The protein(s) responsible for the SEV1 induced pyramids is currently unknown (Wang et al., 2018). However, SEV1 does not encode a SIFV1 gp43 homolog (Baquero et al., 2021).

### 2.7. STIV1 hijacks host cell division machinery during infection

Like many eukaryotic enveloped viruses, STIV1 appears to manipulate its host’s cell division machinery during infection. The Endosomal Sorting Complex Required for Transport (ESCRT) is a system in eukaryotes that is responsible for the formation of multi-vesicular bodies and secretory bodies, and potentially aids in cell division (Schiel and Prekeris, 2010). More recently, members of this system have been discovered to have homologs in the Sulfolobales order (Hobel et al., 2008; Lindås et al., 2008; Samson et al., 2008, 2011; Härtel and Schwille, 2014; Blanch Jover et al., 2022). In their native contexts, the crenarchaeal ESCRT-like proteins CdvA (*S. solfataricus* SSO_0911; no eukaryotic homolog), CdvB (SSO_0910, SSO_0451, SSO_0881, and SSO_0619; Vps24), and CdvC (SSO_0909; Vps4) are responsible for the bilateral fission mechanism that Crenarchaea adopt for cell division, a process likely to be orthologous to that of vesicle formation in eukaryotes (Lindås et al., 2008; Blanch Jover et al., 2022; Blanch Jover and Dekker, 2023). It is thought that CdvA associates with the segregating genomes and then acts as a membrane anchor to recruit and align CdvB and its paralogs, which polymerize to form a contractile ring around the midline of the cell (Moriscot et al., 2011). Depolymerization of the CdvB subunits, catalyzed by CdvC, generates tension in the ring and constricts the cell until it is cleaved into two daughter cells. Several lines of evidence support the involvement of the host ESCRT machinery during STIV1 replication (Figure 3).

For one, the genes of the Cdv system are differentially expressed during STIV1 infection. Transcripts of both CdvA and CdvC, as well as two of the four paralogs of CdvB, are upregulated in infected *S*. *sofataricus* cells (Ortmann et al., 2008). Concordantly, proteomic evidence supports over-expression of CdvA and two of the CdvB paralogs during STIV1 infection (Maaty et al., 2012a,b). Second, there is strong evidence of direct physical associations between Cdv and viral components (Table 2). This includes the presence of a CdvB paralog (SSO_0881) in purified STIV1 virion samples, as measured by mass spectrometry (Maaty et al., 2006). The Cdv system also seems to be directly involved with the lysis pyramids, as CdvC colocalizes with C92 to the sites of pyramid formation and CdvB interacts with C92 in a yeast-two-hybrid assay (Snyder et al., 2013a,b). It is thought that C92 may act as a CdvA analog to recruit CdvB and CdvC, potentially to signal or even catalyze the opening of the lysis pyramids (Table 2; Snyder et al., 2013b). This interaction between the ESCRT system and a virus has been extensively observed in eukaryotic viruses, such as in the budding mechanism of HIV-1 (Schmidt and Teis, 2012). Yeast-two-hybrid assays have also detected interactions between the CdvB paralog SSO_0619 and the MCP B345, which supports a role for CdvB in organizing the assembly of the capsid-membrane complex (Snyder and Young, 2011; Snyder et al., 2013b).

**Table 2:** Description of cellular ESCRT-related components in STIV1 virion assembly and virus-induced lysis.

	**CdvA**	**CdvB**	**CdvC**
*Sulfolobus* gene(s)	SSO 0911	SSO 0910, SSO 0451, SSO 0881, and SSO 0619	SSO 0909
Eukaryotic homolog	None	ESCRT-III	Vps4 ATPase
Functional mechanism	Membrane anchor; interacts with CdvB via E3B domain	Protein forms a filament to promote membrane deformation in cell division and vesicle formation	Depolymerizes the ESCRT-III filaments
*Sulfolobus* cell division	CdvA serves to recruit CdvB protein to center of cell (1, 3)	Protein oligomerizes to form rings in center of cell; involved in membrane constriction (2, 3)	ATPase responsible for depolymerizing the CdvB rings (2, 3)
STIV1 virion assembly	C-terminal end of B345 interacts with CdvB paralog	SSO 0619 interacts with B345 (4); interaction is abolished when C-terminal tail is deleted	--
STIV1-induced cell lysis	C92 acts as a CdvA analog (4)	SSO 0910 interacts with C92 (4)	SSO 0909 is localized to pyramid structures during infection (4)

Cdv, cell division; SSO, *Sulfolobus solfataricus*; E3B, ESCRT-III binding domain.

References: ^1^Samson et al. (2011), ^2^Samson et al. (2008), ^3^Blanch Jover et al. (2022), ^4^Snyder et al. (2013b).

### 2.8. STIV attachment to its host

**Figure 5:**
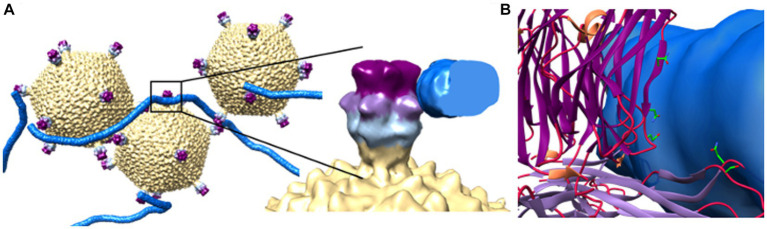
Initial viral attachment to pili. **(A)** Subtomographic average of STIV virions and pili show pilus recognition is mediated by interactions with the 2nd (light purple) and 3rd (dark purple) jelly roll domains of the C381 turret protein. To the right, an enlarged view of a single turret/pilus interaction. **(B)** Strictly conserved, surface exposed residues lie within these regions and potentially mediate recognition. These include Asn196 and Ser215 in domain 2, and Glu285 and Asn289 in domain 3. Adapted from Hartman et al. (2019).

Recently, initial attachment of STIV1 virions to the surface of *S. solfataricus* was studied by CET, wherein, following a 10 min incubation, virions were observed bound to host pilus structures (Figures 3, 5A; Hartman et al., 2019). Subtomographic averaging and docking the atomic model of STIV1 into the resulting density produced a pseudo-atomic resolution structure, showing that the interaction was mediated by conserved, solvent-exposed residues in the second and third domains of the C381 turret protein (Figures 5A,B). Subsequent stages leading to genome entry remain to be studied. Notably, the STIV petal protein (C557), if present, would occlude the pili binding site, suggesting that the petal bound form would be non-infectious. Therefore, C557 may function as a maturation protein, preventing immediate re-adsorption of STIV as it’s released from an infected cell (Hartman et al., 2019). Because C557 interacts with A223 and C381, it might also serve a scaffolding protein function during turret assembly (Hartman et al., 2019).

Initial binding to cellular appendages such as type IV pili and flagella are an emerging theme for archaeal viruses (Hartman et al., 2019). For example, the rudivirus SIRV2 binds to the tip of an unknown type IV pili-like structure (Deng et al., 2014; Quemin et al., 2018). At later time points, however, SIRV2 is found in bits and pieces localized on the cell surface. Like STIV, how SIRV2 migrates from the tip of the pili to the cell surface remains unknown.

### 2.9. Additional non-structural proteins

*CRISPR/Cas and anti-CRISPRs* - The B116 gene product has also been subjected to structural studies, revealing a dimeric protein with a novel fold (Table 1; Larson et al., 2007b). Conserved residues within a large, saddle-shaped cleft suggested the presence of a ligand binding site that could accommodate two-fold symmetric nucleic acid polymers, such as dsDNA. It was suggested that B116 might thus serve to regulate gene expression, or play a role in the synthesis, packaging, or modification of nucleic acid. On the other hand, B116 shares sequence homology to genes in three additional archaeal virus families, *Rudiviridae* (SIRV1, SIRV2, and ARV1), *Lipothrixviridae* (SIFV and AFV1) and *Bicaudaviridae* (ATV) (Larson et al., 2007b). The conservation of B116 across these remarkably different viral families suggests that, instead of a virus specific function, the B116 family of proteins might interact with some common host machinery. Indeed, recent work from Athukoralage et al. (2020) showed that the SIRV1 homolog (gp29) is an anti-CRISPR “ring” nuclease that cleaves cyclic tetra-adenylate (cA_4_) (Figure 3). This four-base cyclic RNA is a second messenger in type III CRISPR-Cas systems that is produced in response to viral infection. cA_4_, in-turn, activates non-specific nucleases to induce cell dormancy (Kazlauskiene et al., 2017; Niewoehner et al., 2017), while potentially stimulating spacer acquisition and activity of the type I CRISPR surveillance complex (aCascade) (Charbonneau et al., 2021). Thus, virally encoded CRISPR ring nucleases (Crn) such as STIV B116 and SIRV gp29 allow viruses to circumvent type III CRISPR defense systems (Larson et al., 2007b; Athukoralage et al., 2020).

*A putative transcriptional regulator* - Structural studies of STIV F93 revealed clear structural homology to the MarR family of DNA binding proteins in bacteria (Table 1; Larson et al., 2007a). Members of the MarR family typically function as transcriptional regulators, but a target has yet to be identified for F93 (Larson et al., 2007a). Bioinformatics analysis clearly indicates the presence of additional transcription factor-like proteins. While some will undoubtedly regulate expression of the viral genome, it is likely that others will orchestrate expression of host genes as well.

### 2.10. Culturing and genetic systems

Much has been learned from structural studies into STIV, but many important questions about how it replicates require other methods of investigation. First, a culturing system was developed for laboratory propagation (Ortmann et al., 2008), and then a genetic system was created for mutational analyses (Wirth et al., 2011). During the establishment of a culturing system for STIV1, numerous *Sulfolobus* species were tested for susceptibility to the virus via transfection and infection assays (Wirth et al., 2011). After repeated rounds of selection and isolation, a highly susceptible *S. solfataricus* P2 variant (P2-2-12 or P2^3^) was found, though, interestingly, limited sequence data was unable to detect any differences between P2^3^ and the original strain. This culturing system led to the development of an STIV plaque assay (Ortmann et al., 2008) and a microarray analysis of *Sulfolobus* gene expression during STIV1 infection (Ortmann et al., 2008). It also led to the creation of a genetic system, composed of a set of subclones and a shuttle vector. The 17 kbp genome of STIV presents certain challenges for molecular methods, and so it was divided into five, slightly overlapping fragments, each of which was cloned into a vector backbone and transformed into *E. coli* (Wirth et al., 2011). With this system, genetic manipulations can be introduced into a subclone, which can then be ligated into the shuttle vector via unique restriction sites and transfected into the host. Mutations introduced into several viral genes have elicited informative phenotypic changes, such as the essentiality of the glycosyl transferase A197, but a great deal of potential insight remains untapped (Table 1).

## 3. STIV2

### 3.1. A sister lineage cut short

Several years after the discovery of STIV1, a sister virus was detected from an enrichment culture collected from Icelandic hot springs (Happonen et al., 2010). Its host was found to be most closely related to *S. islandicus* by 16S rRNA sequence analysis, and its virions were nearly identical to that of STIV1 (Happonen et al., 2010). The genome of STIV2, at 16.6 kbps, was slightly shorter than STIV1, and was estimated to contain 34 ORFs (as opposed to 38 ORFs in STIV1). The behavior of STIV2 and its host suggested a lysogenic phase, as, even with successive single colony isolations, the host continued to produce viable virions and could not be cured of the virus (Happonen et al., 2010). The presence of virion production during these passages was even confirmed with imaging from thin cell sections using transmission electron microscopy (TEM) (Happonen et al., 2010).

Sadly, this variant was only studied for a short time before it could no longer be propagated. Still, valuable information was gleaned from this second member of the *Turriviridae* family through sequencing and structural imaging efforts (Happonen et al., 2010). Genome alignment between STIV2 and STIV1 demonstrates strong homology (25/34 ORFs) across most of the genome, with nucleotide identities in excess of 70% in some homologs, including the MCP (A345 in STIV2). As with STIV1, STIV2 possesses the same prominent turrets, though undecorated with the large petals of STIV1. Interestingly, the turrets also differ in the apparent central channel, which appears larger and more continuous in STIV2. This led the authors of that work to also entertain its role in packaging the viral genome, though this model has since been revised (Happonen et al., 2010). More in depth study into the dsDNA NTPase B204 (B204 in STIV1) produced evidence of a hexameric configuration that could translocate dsDNA molecules through its central pore, powered by NTP hydrolysis (Happonen et al., 2013). Rather than the turret as a site of genome packaging, it was proposed that this protein complex would occupy a unique vertex on the procapsid and catalyze packaging. Still, no direct evidence of this unique vertex exists. Further, the vertices adopt a pentameric geometry, and it is unclear how a hexameric complex would stably occupy such a site.

## 4. STIV3

### 4.1. The replication cycle expands

The third member of the *Turriviridae* family was found not as a free, infectious virion, but integrated into the host genome as a provirus. In early 2016, researchers from the University of Illinois at Urbana-Champaign sequenced the genomes of 47 *S. acidocaldarius* isolates collected from Gibbon Geyser and Norris Geyser basins located in Yellowstone National Park, United States. In 21 of the strains, a 17.1 kbp insert was detected that had high similarity to the STIV1 genome (Anderson et al., 2017). This discovery was quite surprising on two accounts. First, STIV1 (and STIV2 by association) is thought to be a lytic virus, with no known instance of integration in the host genome (Rice et al., 2004; Wirth et al., 2011; Snyder et al., 2011b). Second, STIV1 had never been successfully propagated in a host other than *S. solfataricus*, even when assayed directly against *S. acidocaldarius* (Wirth et al., 2011).

Intriguingly, all three STIV variants harbor putative integrase genes (STIV1, A510; STIV2, B509; and STIV3, B510), which exhibit compelling sequence homology with a diverse class of tyrosine recombinases (Happonen et al., 2010; Maaty et al., 2012a; Overton, 2019). These STIV integrases appear to be related to both the *Sulfolobus* spindle-shaped virus 1 (SSV1) gene, D-335 (Muskhelishvili, 1993; Muskhelishvili et al., 1993; Eilers et al., 2012; Zhan et al., 2012), but even more closely to the bacteriophage *λ* class of integrases (Badel et al., 2021). These recombinases are structured such that the N-terminus serves as both a multimerization site and to bind the target DNA sequence, and the C-terminus catalyzes recombination between the viral and host genomes at attachment sites attP and attB, respectively (Zhan et al., 2012; Badel et al., 2021). Proteins in this group can adopt other functions as well, such as resolving DNA supercoiling during synthesis and resolving concatemers in certain viral replication strategies (Esposito and Scocca, 1997; Jayaram et al., 2015). By investigating the STIV-like provirus in *S. acidocaldarius* strains, we anticipate the addition of an entirely new facet of the STIV infection cycle.

The presence of a STIV3 provirus suggests that B510 (A510 in STIV1) does function as an integrase. The lack of stop codon accumulation, minimal dN/dS values, and perfectly conserved catalytic domain residues suggest that this gene is functional in the other variants as well (Overton, 2019). When aligned with several other tyrosine recombinases, the active site motif R-X_n_-[R/K]-X_n_-[H/K]-X_n_-R-X-X-[R/H/W]-X_n_-Y (Esposito and Scocca, 1997) is perfectly conserved across the variants. Several other residues were well conserved across all recombinases, pointing toward other potentially critical residues. Integrated proviruses have not been specifically probed in STIV1, but perhaps this should be revisited, especially in the original host isolate. It may be the case that the highly susceptible host strain P2^3^ is resistant to viral integration, which is supported by the absence of the canonical CCTAGG att site, and this forces a more proliferative transmissive propagation by denying the virus its lysogenic option.

It should be noted that the initial isolation of STIV1 was achieved through multiple rounds of host cell isolation, which would seem to exclude free virions, and instead support the carry-over of temperate or integrated virus (Rice et al., 2004). The same procedure was employed in the isolation of STIV2 (Happonen et al., 2010). Further, strong lytic replication in STIV1 was only observed after selecting for a highly susceptible host (Ortmann et al., 2008). Many viruses that infect thermophilic archaeal hosts are temperate (Dellas et al., 2014), and the characterization of STIV1 as lytic only was surprising at the time of its discovery (Rice et al., 2004). All three STIV variants possess an identical CCTAGG att sequence at the same position in the genome, and so, from this aspect, should be capable of integration (Figure 3). All of this raises some intriguing possibilities, but the STIV3 system is still new and understudied, and so they have yet to be investigated.

## 5. Many facets of STIV remain elusive

### 5.1. How does STIV enter its host?

While there is significant insight into initial attachment (Hartman et al., 2019; discussed above), how the STIV genome subsequently enters the cell is largely speculative. Viruses generally accomplish this by either direct injection, or in the case of enveloped viruses, membrane fusion. One proposed function for the turret channels observed in STIV1 and STIV2 is to inject the genome directly into the host (Maaty et al., 2006). However, while the turrets are ~12 nm in length, the host cell membrane is encased within a 40 nm thick, semi-crystalline, proteinaceous S-layer, that is itself heavily glycosylated with extended (~30 nm) carbohydrate chains (Hartman et al., 2019). A direct injection model would thus require STIV to first penetrate the glycan barrier and subsequent S-layer. Logically, it would also require the ability to form a channel through the membrane, a function that is unknown for the viral jelly roll fold. From this perspective, direct injection through the turret protein seems unlikely.

Another possibility is that the viral capsid partially disassembles near the exterior surface of the host cell, and A55 or other unidentified proteins embedded in the internal lipid membrane might then direct genome injection or membrane fusion. Viral disassembly is a common mechanism in eukaryotic viruses (Quemin et al., 2018), and in the case of STIV may be supported by the inferred instability of B345 multimers. B345 crystallizes as a monomer rather than any of the higher order oligomers present in the assembled capsid. This suggests the capsid could disassemble under the right conditions (Khayat et al., 2010; Hartman et al., 2019). In this light, potential disassembly intermediates have been observed (Hartman et al., 2019), but how genome injection or membrane fusion occurs remains to be determined. In either case (injection or fusion), however, the STIV virion would still need to penetrate the glycan barrier and S-layer.

Another possible mechanism involves the pili structures that drive cell adhesion and biofilm formation in *Sulfolobus* species. Bacterial Type IV pili, homologs to the archaeal type IV pili, are hijacked by other viruses, such as the RNA bacteriophage of *Pseudomonas aerugenosa*. In this case, the virion anchors itself to the bacterial pilus and induces its retraction, pulling the hitchhiking virus into close proximity to the cell surface (Bradley, 1972). If the pili observed in *S. solfataricus* share in this capacity for retraction, STIV could exploit it to move through the S-layer barrier to the membrane. Intriguingly, retraction of type IV pili in *S. acidocaldarius* has recently been observed, though this activity has yet to be demonstrated in *S. solfataricus* (Charles-Orszag et al., 2023). Conversely, even a biased random walk along a tangle of pili could draw the STIV virions toward a similar result. This, in combination with B345 dissociation, might then provide the contact between the viral and cellular membranes needed for membrane fusion or direct injection. For these reasons, these later time points in STIV attachment and entry are of great interest, especially since the cyclic tetraether lipid monolayer of the viral envelop appears to be incompatible with the hemifusion intermediate of the canonical membrane fusion model.

### 5.2. How is the internal viral envelope acquired?

Most enveloped viruses acquire their envelope from the cellular plasma membrane (or another intracellular membrane structure, such as the Golgi or Endoplasmic Reticulum) upon egress in a process referred to as budding. However, unlike other archaeal viruses such as SSV1, STIV viruses do not bud from their hosts (Palm et al., 1991; Maaty et al., 2006). In fact, the membrane of STIV contains only a subset of the lipid moieties present in the host membrane, which suggest that STIV virions must obtain their envelopes in the cytoplasm. The mechanism that STIV utilizes to acquire this envelope has yet to be elucidated, but may be similar that of some bacteriophages. The bacteriophage phi6 utilizes cytoplasmic membrane vesicles for its envelope (Johnson and Mindich, 1994; Mäntynen et al., 2019); however, phi6 contains an external membrane, as opposed to an internal membrane as seen in STIV viruses. Another internal envelope-containing virus, PRD1, contains phage-encoded membrane proteins that are recruited into the cellular plasma membrane during viral infection (Mindich et al., 1982; Mäntynen et al., 2019). The viral membrane is then acquired by the phage in a process that mimics clathrin-coated endocytosis in eukaryotic cells (Rydman et al., 2001; Mäntynen et al., 2019).

The acquisition of the internal STIV envelope may involve components of the cellular ESCRT machinery. As previously mentioned, yeast-two-hybrid experiments have detected an interaction between CdvB (SSO_0619) and STIV1 B345 (MCP) (Snyder et al., 2013a). In this context, CdvB could be serving a role in recruiting and organizing B345 (MCP) to internal membranes that will eventually form the capsid-membrane complex. In addition, membrane vesicles released by some *Sulfolobus* species have been found to contain components of the cellular ESCRT system (Ellen et al., 2009).

### 5.3. Is STIV lysogenic?

Previous work has suggested that STIV1 only employs the lytic cycle in its replication (Rice et al., 2004; Wirth et al., 2011), which seemed all the more probable given its striking lysis system (Snyder et al., 2011b), and that it was expressed early in the infection cycle (Ortmann et al., 2008). Paradoxically, the gene A510 appears to be a recombinase and is expressed by the cell during STIV1 infection (Maaty et al., 2012a). Further, the STIV1 genome contains an att site at the same location as the att site in STIV3. However, there is not an att site present in sequenced *S. solfataricus* genomes. Therefore, the use of a *S. solfataricus* host for STIV1 propagation may have prevented the detection of lysogeny. Unfortunately, no additional investigations have been performed to reconcile this apparent contradiction. A potential alternative role for A510 could come in the resolution of genome concatemers produced during rolling circle replication. This mechanism has not been directly observed in STIV1 but is employed in many other virus systems (Better and Freifelder, 1983; Kusumoto-Matsuo et al., 2011).

### 5.4. How does STIV package its genome?

Evidence to date seems to support a genome packaging mechanism analogous to that of some dsDNA viruses such as Herpesvirus, which passes its genome through a unique portal vertex, powered by an ATPase (Hong et al., 2014). While a similar unique portal has not been observed in structural STIV1 studies, there is strong evidence that the DNA packaging ATPase, B204, transports the genome as a linear dsDNA molecule into the capsid (Maaty et al., 2006; Happonen et al., 2013; Dellas et al., 2016). Still, given the evidence that the STIV1 genome is circular, questions remain about how the linearized genome would ligate into a circular one inside the capsid. Further, the host derived DNA-binding protein, SSO7D, known for its promiscuous activities in DNA binding, RNA cleavage, and protein disaggregation, has been detected in purified virions (Maaty et al., 2006). How does this protein get into the capsid? Is it bound to the DNA prior to transport, or is it incorporated into the capsid before DNA packaging? And what, specifically, is its function in STIV packaging or replication?

### 5.5. What is the function of the viral lattice structures and how are they formed?

The quasi-crystalline viral lattices that assemble within the cell toward the end of the STIV1 infection cycle also elicit questions about the organizing principle behind them, and what function they serve the virus. Since the population of capsids inside the lattice is predominantly filled, while that of the diffuse capsids is mostly empty, it may serve as a microenvironment to organize genome packaging (Fu et al., 2010; Fu and Johnson, 2012). Alternatively, or in addition, such organization might offer an important advantage in that it accommodates the greatest number of viruses within the limited cellular space. In this context, genome packaging and maturation may prime the capsid to interact with other mature virions, and so catalyze lattice formation. It has been suggested that the C557 petals that often adorn STIV1 may act to protect the turrets of nascent mature virions from blindly sticking to the freshly lysed host cell pili. This situation would trap the virions in a dead end (Hartman et al., 2019). However, it is surprising that STIV1 should carry such a genomic load (the C557 ORF makes up 9.5% of the viral genome [1,671 bps out of 17,663 bps total]) for a function that could be performed by a protein half the size, that is, unless it has a second role. It seems likely that the petals are attached during virion assembly, before or during lattice formation. Therefore, the petals may act to orient the virions within the quasi-crystalline array. Once the pyramids burst open and the virions are carried out of the cell with the leaking cytoplasm, dilution into the acidic extracellular environment likely causes C557 to dissociate, but perhaps not before clearing the ensnaring pili of the dead host cell (Hartman et al., 2019). Regardless of the true origin and function of these virus lattices, this facet of the STIV infection cycle begs for more study.

### 5.6. What triggers the pyramidal lysis system?

The STIV lysis system is a stunning example of biological ingenuity, to take a 92-residue peptide and produce such an elegant and effective structure. While some aspects of this system have been worked out, from the geometric details of the pyramids to some of the molecular interactions that impart their shape, the real crux of the system, molecular events that trigger the moment of lysis, have yet to be determined. One proposed mechanism is that, along with the forces acting on the pyramid walls to push them outward from the cell envelope, there are torsional forces that build in the pyramid structure as well (Daum et al., 2014). The observation of a slight curvature of the pyramid vertices just prior to opening and a twisted, outward curling of the facets after opening are offered as evidence for this torsional force. If this is the case, then, perhaps after a triggering event, the facets could be ripped along the vertex seams by the torsional forces, and this is what catalyzes pyramid lysis. Still, the observed curvatures of the pyramid vertices are quite subtle and not consistent, though the curling of the petals is apparent. As well, pyramids have not yet been captured in the process of opening, and so evidence for a mechanical explanation is hard to come by. Another possible mechanism is the catalysis of pyramid edge unzipping by the Cdv system, particularly CdvC. The fact that Cdv proteins interact with C92 and that CdvC localizes to the pyramids, as well as the established depolymerization activity of CdvC, support this argument. Thankfully, molecular methods exist to test this proposed mechanism, such as Cdv gene knockdown and mutagenesis experiments. Another, related open question is what factors regulate the size of the pyramids. They grow to roughly the same size in completely heterologous species, and so the involvement of a specific host factor seems unlikely (Daum et al., 2014). If it is found that C92, itself, controls pyramid size, this protein will become an even more powerful example of biological ingenuity and multitasking.

### 5.7. What drives the dispersal of STIV, and does its host play a role?

Metagenomic evidence suggests that sympatric viruses to STIV disperse extensively at the local scale, and the detection of viral sequences harvested from the surrounding air implicate water vapor as a vehicle for aerial transport (Snyder et al., 2007). There is in fact a large body of evidence that not only virions, but larger classes of microbes such as bacteria, fungal spores, and pollen move freely through the air, and can even ride atmospheric currents to disparate locations across the planet (Kellogg and Griffin, 2006; Griffin, 2007). However, we do not yet know how robust STIV virions are to environmental stresses. It would seem that STIV would have to evolve an extreme structural stability to survive in the heat and acidity of the native environment, even for a short time. It may also be intuitive to think that a highly stable shell would be even more stable under “less hostile” conditions like a water droplet in the ambient air. Yet, highly stressful conditions may require highly specific solutions, and under what we consider permissive environments may be anything but for the inhabitants of acidic hot springs. The evidence that members of the STIV family are able to move in and out of the host genome even opens up the possibility of genomic hitchhiking, in which a provirus could be transported safely in the host cell during aerial transport. There is, obviously, a great deal more to explore. Still, it is fascinating to imagine the dynamics and scale with the understanding that the *Sulfolobus*/STIV system may be a planet-wide phenomenon, which could harbor whole new coevolutionary processes. This begs for more extensive sampling, both geographically and temporally, and new investigations into the dynamics and mechanisms that drive this complex coevolutionary dance.

## 6. Current studies and future endeavors

Currently, our work is focused on the development and validation of a genetic system for STIV3 and its *S. acidocaldarius* host, as well as experiments to efficiently induce the STIV3 provirus from the host for viral propagation and study of the lysogenic cycle. This will be coupled with mutagenic studies on the B510 gene (STIV3 integrase) and *att* sites to further elucidate the mechanisms of viral integration and the regulatory factors that dictate its behavior. Looking further ahead, a highly promising application of this system is the study of the interactions between C92 and B345 in STIV3, and the ESCRT system in the host. Mutagenic studies on each could elucidate key factors and residues involved in their associations. In addition, we plan to conduct metagenomic comparisons of environmental samples to detect the conservation of core genes in natural communities.

It is clear from our research, and the published literature, that there is much left to study in the STIV system, and in archaeal viruses in general. Even with the extensive investigations into the gene expression, lytic system, and ESCRT interactions, we still do not have a comprehensive model of the infection cycle. The primary reason for this is that STIV viruses were discovered only recently, and few labs intensively study this system. An impressive amount of knowledge has been generated through the study of STIV1 (Figure 3), and we expect STIV3 will be just as ripe for discovery. We hope that others share in our curiosity about this enigmatic virus family.

## Author contributions

MO: Writing – original draft, Writing – review & editing. RM: Writing – original draft, Writing – review & editing. CL: Writing – review & editing. JS: Conceptualization, Writing – original draft, Writing – review & editing.

## Funding

The author(s) declare that no financial support was received for the research, authorship, and/or publication of this article.

## Conflict of interest

The authors declare that the research performed for writing this review was conducted in the absence of any commercial or financial relationships that could be construed as a potential conflict of interest.

The author(s) declared that they were an editorial board member of Frontiers, at the time of submission. This had no impact on the peer review process and the final decision.

## Publisher’s note

All claims expressed in this article are solely those of the authors and do not necessarily represent those of their affiliated organizations, or those of the publisher, the editors and the reviewers. Any product that may be evaluated in this article, or claim that may be made by its manufacturer, is not guaranteed or endorsed by the publisher.
